# Providers and women’s perspectives on person-centered maternity care: a mixed methods study in Kenya

**DOI:** 10.1186/s12939-019-0980-8

**Published:** 2019-06-10

**Authors:** May Sudhinaraset, Katie Giessler, Ginger Golub, Patience Afulani

**Affiliations:** 1 University of California, Los Angeles, Jonathan and Karin Fielding School of Public Health, Los Angeles, USA; 2 School of Medicine, University of California, San Francisco, USA; 3 Innovations for Poverty Action, Nairobi, Kenya

**Keywords:** Quality of care, Person-centered care, Health communication, Health facility, Kenya, Provider perspective, Women perspective, Childbirth, Delivery, Labor

## Abstract

**Background:**

Globally, there has been increasing attention to women’s experiences of care and calls for a person-centered care approach. At the heart of this approach is the patient-provider relationship. It is necessary to examine the extent to which providers and women agree on the care that is provided and received. Studies have found that incongruence between women’s and providers’ perceptions may negatively impact women’s compliance, satisfaction, and future use of health facilities. However, there are no studies that examine patient and provider perspectives on person-centered care.

**Methods:**

To fill this gap in the literature, we use cross-sectional data of 531 women and 33 providers in seven government health facilities in Kenya to assess concordance and discordance in person-centered care measures. Additionally, we analyze 41 in-depth interviews with providers from three of these facilities to examine why differences in reporting may occur. Descriptive statistical methods were used to measure the magnitude of differences between reports of women and reports of providers. Thematic analyses were conducted for provider surveys.

**Results:**

Our findings suggest high discordance between women and providers’ perspectives in regard to person-centered care experiences. On average, women reported lower levels of person-centered care compared to providers, including low respectful and dignified care, communication and autonomy, and supportive care. Providers were more likely to report higher rates of poor health facility environment such as having sufficient staff. We summarize the overarching reasons for the divergence in women and provider reports as: 1) different understanding or interpretation of person-centered care behaviors, and 2) different expectations, norms or values of provider behaviors. Providers rationalized abuse towards women, did not allow a companion of choice, and blamed women for poor patient-provider communication. Women lacked assurance in privacy and confidentiality, and faced challenges related to the health facility environment. Providers attributed poor person-centered care to both individual and facility/systemic factors.

**Conclusions:**

Implications of this study suggests that providers should be trained on person-centered care approaches and women should be counseled on understanding patient rights and how to communicate with health professionals.

## Background

Alarming rates of poor patient experiences, particularly in developing contexts, include high prevalence of mistreatment of women [1] and disrespect and abuse [2, 3], such as being hit, slapped, verbally abused, not consenting to procedures, and abandonment during childbirth [1]. Consequently, global movements have called for the need to focus on person-centered maternity care (PCMC) [4]. PCMC is a key domain of quality of care that emphasize care that is respectful of and responsive to women’s preferences and needs [5, 6]. It captures the interpersonal dimensions of care and the need to involve women in their care to improve the quality of patient experiences. Domains of PCMC include dignity and respect, communication and autonomy, and supportive care. Importantly, patient-provider relationships are at the heart of person-centered care, with patient trust in providers leading to greater adherence to treatments, continuity of care, and increased patient satisfaction [7].

The issue of quality of care, including PCMC, is especially relevant and timely in Kenya given high levels of maternal deaths and low quality of care. The Kenyan Government recently released a report indicating that 90% of the 484 maternal deaths at major referral hospitals in 2017 resulted from poor quality of care [8]. In addition, one out of five women report feeling humiliated during labor and delivery [9], 8.5% report non-confidential care, 18% report non-dignified care, 14.3% report neglect or abandonment, 4.3% report non-consensual care, 4.2% report physical abuse, and 8.1% report detainment for non-payment of fees. Consequently, there is a renewed commitment in the country to identify actionable strategies to improve the survival of mothers and newborns and address gaps in health systems. Despite this progress, concerted efforts are needed to improve women’s negative experiences during childbirth. Providing person-centered care and meeting the cultural preferences, needs, and values of a diverse population is therefore critical in improving maternal and newborn health.

Literature from outside the maternal health field suggests that incongruence between women’s and providers’ perceptions and viewpoints may negatively impact women’s compliance, satisfaction, and future use of health facilities [10]. While providers may place an emphasis on clinical quality, past studies find that marginalized communities are more likely to judge their care based on non-discriminatory practices and non-medical expectations [11]. Flickinger et al. [12] found that clinicians who had higher respect for their patients tended to engage in more “rapport building, social chit chat, and positive talk” and thus provided more patient-centered care with less “clinician verbal dominance”.

While a small number of studies on women’s experiences during labor and delivery care include data from both women and providers, most are qualitative in nature [1], and there is a dearth of information that specifically compares the responses of the two populations to assess congruence and discordance in perceptions and drivers of quality. Very few studies have explored providers’ perspectives on PCMC. One recent qualitative study from Kenya among women, providers, and facility staff highlights drivers of mistreatment, including women not understanding their rights, social norms and gender inequalities; and health systems factors such as poor governance and leadership and power differentials between women and providers [13]. Moreover, providers reported physical abuse was justified to save women’s lives and improve the safety of the mother and baby [13]. Understanding the same behaviors from different perspectives will allow us to tailor quality improvement interventions to nurses, doctors, and hospital staff.

This study fills two gaps in the existing literature. First, few studies compare patient and provider perspectives on person-centered care during childbirth. Second, there is a paucity of research on provider perceptions of person-centered maternity care. The objective of this mixed-methods study is to assess person-centered care through the perspective of providers and women. We take advantage of recent progress in defining and measuring person-centered care during childbirth to compare women and providers’ perspectives.

This study uses the PCMC scale developed and validated by Afulani and colleagues [10]. The validation of the scale in Kenya is described in detail elsewhere [6]. The PCMC scale has 30 items with three subscales. The Dignity and respect subscale has 6 questions, including questions such as whether providers demonstrated respect, treated women in a friendly manner, or physically/verbally abused women. The communication and autonomy subscale has six questions capturing the extent to which women were given information and included in their care; and the supportive care subscale has 15 questions capturing indicators of provider and social support and the health facility environment. The scale has been validated in Kenya, India, and Ghana [14, 15].

This study uses the PCMC items for women and applies them to providers to be able to compare specific behaviors across these two populations. We use quantitative data to describe specific person-centered care behaviors across women and providers to assess differences across the two populations. We then specifically focus on qualitative data with providers to identify providers’ description and challenges of providing person-centered care. The paper focuses on providers’ attitudes in qualitative data because they are the likely critical points for change and attention to improve care. Our research questions are twofold: first, do providers and women differ in their perceptions of person-centered care? Second, what are potential reasons for differences in reports of person-centered care from the provider’s perspective?

## Methods

### Study setting

Seven government health facilities within two contiguous, urban/peri-urban counties in Kenya (Nairobi and Kiambu) participated in the study. These facilities include health centers, sub-county hospitals, and district hospitals, in order of increasing size and capability. As part of a larger quality improvement project, the research team identified potential facilities in collaboration with county officials. Facilities were identified based on location (in Nairobi/Kiambu), delivery volume (higher volume facilities), and interest in partaking in the quality improvement project. Three of these facilities were randomly selected to partake in a quality improvement initiative, while three were considered control facilities. Due to budgetary constraints, the seventh facility was not included in the larger quality improvement initiative; however, data was obtained from providers and are therefore included in these analyses. Quantitative data collection for providers and women occurred across all seven facilities, while qualitative interviews were conducted only at intervention facilities.

### Surveys with women: sample and data collection

Exit surveys were conducted with 531 women from August to December 2016. Eligible women were recruited from the hospital until our target sample size was reached. All surveys were administered at a private space within the facility. Prior to data collection, six female enumerators were trained on the survey tool for 1 week, followed by 1 week of piloting at participating facilities. Recruitment was a collaborative effort between the study team and the facility staff who identified potential respondents and directed them to a private area at the facility where the enumerator introduced the study and confirmed eligibility. Eligible respondents were women who 1) were aged 15 to 49 years, 2) delivered within the past 7 days, and 3) owned or had access to a functional cell phone to allow for follow-up. If the woman was eligible and interested, the enumerator sought written informed consent in a language of her preference prior to administering any survey questions.

Enumerators conducted the survey electronically using a tablet with the SurveyCTO application and in the respondent’s preferred language. They collected data on demographics and the level of person-centered maternity care (PCMC) received during delivery. Participants received approximately $1.50 in airtime credit to thank them for their participation in the study. Each day, enumerators sent any surveys conducted to the password protected SurveyCTO server, and it was then downloaded to an encrypted server. Throughout data collection, study staff conducted high frequency checks regularly, as well as back checks via phone, and in-person spot checks and accompaniments for data quality assurance purposes.

### Surveys with providers: sample and data collection

Written informed consent was obtained at the individual and facility level for provider surveys. First, the facility administrator consented to participating in the study. The facility department lead/superintendent then helped the study team to purposively sample potential respondents by staff cadre. In a private space at the facility, interviewers obtained written informed consent for all interested and eligible staff. Consents were obtained prior to beginning any data collection. Respondents received approximately $1.50 in airtime credit to thank them for their participation in the study.

A total of 33 surveys were conducted with maternity providers from the seven study facilities between November 2016 and January 2017. The provider survey tool was developed based on questions from the PCMC scale for interviews with women. Questions were rephrased from the PCMC scale for providers. Instead of asking about women’s specific experiences, questions were rephrased to ask the opinion of providers such as: “Are delivery patients allowed to have someone stay with them during delivery?” “Do you feel the doctors and nurses pay attention to delivery patients during their stay in the facility?” and “Thinking about the labor and postnatal wards, do you feel the health facility is crowded?” Four female enumerators that were previously trained using a similar tool were trained on the survey and piloted the tool at a facility outside the research sample. The questionnaire included questions about their perspective of the facility’s current level of person-centered care for maternity clients, acceptability of various circumstances, job satisfaction, and demographic information. Each day enumerators uploaded the survey responses to a password-protected server, which could only be accessed by the study team, and data were then downloaded to an encrypted server.

### Qualitative interviews with providers: sample and data collection

Qualitative in-depth interviews were conducted in August and September 2016 with maternity providers, non-clinical staff and cleaners from the three intervention facilities. Like provider surveys, the consent process included both the facility and provider levels and followed similar procedures. Respondents for in-depth interviews consented to the interview being audio-recorded. Although they had the option to decline, no respondents did so. Interviewers assured respondents that all interviews would remain anonymous and confidential, and that none of the interview content would be shared with their supervisors or have any effect on their performance evaluation. Interviews were conducted in a private space on the facility grounds to ensure confidentiality. Respondents received approximately $1.50 in airtime credit to thank them for their participation in the study.

A total of 41 in-depth interviews were conducted across three facilities. Thirteen respondents were from facility 1, while 16 were from facility 2 and 12 were from facility 3. The distribution of their position in the facility included 1 clinical officer, 18 maternity doctor/nurses, eight laboratory/pharmacy technicians, and 14 cleaners. A high number of cleaners were included in the study because of past research suggesting mistreatment of women by cleaners in India, South Africa, and Kenya [16, 17]. Therefore, cleaners were included in the study to further explore attitudes for person-centered maternity care. No provider refused to be interviewed.

Participants were also probed about their sense of acceptability with regard to behaviors that contradict foundational elements of person-centered care such as verbal and physical abuse, lack of shared decision-making, and discrimination based on an attribute such as age or religion.

Two female interviewers that were previously trained using a similar tool were trained on this interview guide and piloted the tool in one control facility. Each day, enumerators uploaded the audio recordings to an encrypted server only accessed by the study team. An independent firm was hired to transcribe the recordings verbatim. Simultaneous translation to English was done, where applicable. All transcripts were back checked against at least 50% of the recording to ensure accuracy.

### Data analyses

#### Providers and women quantitative analyses

First, we examined demographic characteristics of all respondents in the individual datasets from the surveys with women and providers. Next, we recoded the PCMC variables from a four-point frequency responses scale to binary responses, coding “no, never and a few times” together and “yes, most of the time and all the time” together. We then renamed the PCMC variables in the provider surveys to be consistent with variables names used in the surveys with women and appended the two datasets for analysis. Finally, we ran descriptive statistics looking at PCMC reports by women and providers. Because of the very small sample size for providers (*N* = 33) relative to women (*N* = 531), we decided not to focus on statistically significant differences between women and provider reports, but rather on the magnitude of the differences.

#### Provider qualitative analyses

Qualitative interviews were analyzed using a Grounded Theory framework and an inductive coding approach [18]. Four researchers initially reviewed the same interview independently to identify common themes related to person-centered care that emerged from the data (i.e. respectful care, informed consent, privacy, communication, etc.). Following comparison and review of independently identified themes generated by the research team, two of the researchers developed a codebook to capture these common themes. A definition for each code was included in the codebook, allowing for standardization of coding across the data. The codebook was then applied to the qualitative interviews using Atlas. ti qualitative analysis software. If and when additional themes emerged during analysis, new codes were created and added to the codebook. Any transcript that had already been analyzed and coded was then reviewed again to ensure new codes were applied if applicable. After all transcripts had been coded, further thematic analysis was conducted to group coded sections of text related to the person-cenetered care themes of dignity, respect, communication, the health facility environment, autonomy and supportive care.

### Ethics, consent and permission

Participants all provided written informed consent in a language of their preference prior to administering any survey questions or interviews. All study documents were reviewed and approved by the ethics review boards at the University of California, San Francisco and the Kenya Medical Research Institute.

## Results

### Demographics for women and providers: quantitative

Tables 1 and 2 show the demographic characteristics of women and providers. The average age was 26 years for women. Approximately 72% of the women were married, with an average of two children. Over 60% had more than a primary education, and most were literate (93%). Most of the women were also in the upper wealth quintiles (96.4%). The average age for providers was 35 years, and 79% were female. Most (94%) were nurses or midwives, with an average of 10 years of experience in their current position.

**Table 1:** Demographic characteristics of survey of women in Nairobi and Kiambu, Kenya

Characteristics	No.	%
Age: Mean (SD)	531	25.6
Parity: Mean (SD)	531	2.1
Marital status		
Single	61	11.5
Partnered/Cohabiting	75	14.1
Married	383	72.1
Widowed	1	0.2
Divorced/Separated	11	2.1
Education		
No school/Primary	204	38.4
Post-primary/vocational/Secondary	242	45.6
College or above	85	16
Literacy: reading		
No, cannot read	2	0.4
Yes, but with some difficulty	33	6.2
Yes, Very well	496	93.4
Literacy: writing		
No, cannot write	3	0.6
Yes, but with some difficulty	29	5.5
Yes, Very well	499	94
Employed		
No	251	47.3
Yes	280	52.7
Wealth Quintile		
Poorest	0	0
Poorer	1	0.2
Middle	18	3.4
Richer	88	16.6
Richest	424	79.8
Religion		
None	2	0.4
Catholic	130	24.5
Protestant	316	59.5
Muslim	5	0.9
Other Christian	78	14.7
Pregnancy complications		
No	447	84.2
Yes	84	15.8
Number of antenatal care visits		
No ANC	225	42.8
Less than 4	248	47.1
4 or 5	53	10.1
Maternity facility type		
Gov’t Hospital	432	81.4
Gov’t Health center	99	18.6
Maternity provider		
Nurse/Midwife	268	50.5
Doctor	147	27.7
Clinical Officer/Medical Assistant	1	0.19
Non-skilled attendant	3	0.56
1plus skilled providers	112	21.1
Maternity Provider sex		
Male	74	13.9
Female	371	69.9
Both	86	16.2
Total	531	100.0

Legend: Household wealth is measured in quintiles calculated from a wealth index based on several questions on household assets in each of the datasets

**Table 2:** Provider Demographics

Characteristics	No.	%
Age: Mean (SD)	33	35.2
Gender		
Male	7	21.2
Female	26	78.8
Position		
Clinical officer	2	6.1
Nurse/Midwife	31	93.9
Years in current position: Mean (SD)	35	10.1
Facility type		
Government Hospital	25	71.4
Government Health Center	10	28.6
Work hours per week: Mean (SD)	33	41.6
Years at current facility: Mean (SD)	35	3.4
Religion		
Catholic	8	24.2
Protestant	22	66.7
Other Christian	3	9.1
Total	33	100.0

### Demographic characteristics for providers: qualitative

Table 3 shows characteristics of maternity providers who participated in the in-depth interviews. Providers interviewed included mostly nurses/midwives (*n* = 18; 43.9%) and cleaners (*n* = 14; 34.2%). Most providers interviewed were female (*n* = 38; 92.7%) with a median age of 36 years. Providers had a median of 3 years work experience with varying levels of educational achievement, though nearly all had received less than a Bachelor’s degree (*n* = 37, 90.2%).

**Table 3:** Maternity Provider Characteristics from Interviews Conducted at Three Public Facilities in Nairobi and Kiambu, Kenya

Characteristics	n	%
Provider/staff title		
Clinical Officer	1	2.4
Nurse/midwife	18	43.9
Laboratory technician	5	12.2
Pharmacists	3	7.3
Cleaner	14	34.2
Age (median)	41	36
Gender		
Female	38	92.7
Male	3	7.3
Religion		
Protestant	27	65.9
Catholic	13	31.7
Seventh Day Adventist	1	2.4
Educational Achievement		
Primary school or below	10	24.4
Secondary school	6	14.6
Diploma (Associates Degree)	21	51.2
Degree (Bachelor)	2	4.9
Masters	2	4.9
Years experience (median)	40	3.0
Working hours/week (median)	41	40.0

### Quantitative results: concordance/discordance of PCMC measures for women and providers

Table 4 shows the distribution of the PCMC variables for women and providers. With a few exceptions, a lower proportion of women reported positively on PCMC domains than providers. For example, among the items in the Dignity and Respect domain, about 86% of women reported they were treated with respect most or all the time, compared to 91% of providers who said they treated women with respect most or all the time. In addition, 83% of women reported they were treated in a friendly manner most or all the time, compared to 94% of providers who said they treated women in a friendly manner most or all the time. Providers were, however, more likely to report verbal and physical abuse than women, with 18 and 2% of women reporting some verbal and physical abuse respectively, compared to 42 and 6% respectively of providers. Women were more likely to report lack of visual privacy than providers, but reports of confidentiality were similar.

**Table 4:** Distribution of PCMC variables for women and providers

		Woman		Provider	
Domain	Item	No.	%	No.	%
Dignity and respect	Treated with respect				
	A few times or never	77	14.5	3	9.1
	Most or all the time	454	85.5	30	90.9
	Friendly				
	A few times or never	89	16.8	2	6.1
	Most or all the time	442	83.2	31	93.9
	Verbal abuse				
	Never	435	82.1	19	57.6
	At least once	95	17.9	14	42.4
	Physical abuse				
	Never	520	98.1	31	93.9
	At least once	10	1.9	2	6.1
	Visual privacy				
	A few times or never	251	47.3	9	27.3
	Most or all the time	254	47.8	24	72.7
	Not applicable	26	4.9	0	0
	Record confidentiality				
	A few times or never	79	14.9	5	15.2
	Most or all the time	452	85.1	28	84.8
Communication and autonomy	Introduce self				
	A few or none of them	494	93.0	27	81.8
	Most or all of them	37	7.0	6	18.2
	Called by name				
	A few times or never	363	68.4	3	9.1
	Most or all the time	168	31.6	30	90.9
	Involvement in care				
	A few times or never	91	17.1	2	6.1
	Most or all the time	302	56.9	31	93.9
	Not applicable	138	26.0	0	0
	Consent to procedures				
	A few times or never	277	52.2	2	6.1
	Most or all the time	254	47.8	31	93.9
	Delivery position choice				
	A few times or never	256	48.2	31	93.9
	Most or all the time	275	51.8	2	6.1
	Language				
	A few times or never	8	1.5	2	6.1
	Most or all the time	523	98.5	31	93.9
	Explain exams/ procedures				
	A few times or never	190	35.8	1	3.0
	Most or all the time	341	64.2	32	97.0
	Explain medicines				
	A few times or never	163	30.7	0	0
	Most or all the time	224	42.2	33	100.0
	Not applicable	144	27.1	0	0
	Able to ask questions				
	A few times or never	193	36.3	2	6.1
	Most or all the time	338	63.7	31	93.9
Supportive Care	Time to care				
	very or somewhat short	391	73.6	27	81.8
	very or somewhat long	140	26.4	6	18.2
	Labor support				
	A few times or never	260	49.0	29	87.9
	Most or all the time	34	6.4	4	12.1
	Not applicable	237	44.6	0	0
	Childbirth support				
	A few times or never	251	47.3	31	93.9
	Most or all the time	22	4.1	2	6.1
	Not applicable	258	48.6	0	0
	Talk about feeling				
	A few times or never	210	39.5	7	21.2
	Most or all the time	321	60.5	26	78.8
	Support anxiety				
	A few times or never	190	35.8	6	18.2
	Most or all the time	186	35.0	27	81.8
	Not applicable	155	29.2	0	0
	Attention when need help				
	A few times or never	127	23.9	0	0
	Most or all the time	404	76.1	33	100
	Took best care				
	A few times or never	82	15.4	2	6.1
	Most or all the time	449	84.6	31	93.9
	Control pain				
	A few times or never	300	56.5	11	33.3
	Most or all the time	231	43.5	22	66.7
	Trust				
	A few times or never	72	13.6	0	0
	Most or all the time	459	86.4	33	100.0
	Enough staff				
	A few times or never	135	25.4	32	97.0
	Most or all the time	396	74.6	1	3.0
	Crowded				
	A few times or never	336	63.3	19	57.6
	Most or all the time	195	36.7	14	42.4
	Cleanliness				
	Very dirty or dirty	500	94.2	1	6.1
	Very clean or clean	31	5.8	32	93.9
	Water				
	A few times or never	40	7.5	5	15.2
	Most or all the time	491	92.5	28	84.8
	Electricity				
	A few times or never	15	2.8	1	3.0
	Most or all the time	516	97.2	32	97.0
	Safe				
	A few times or never	35	6.6	1	3.0
	Most or all the time	496	93.4	32	97.0
	Total	531	100.0	33	100.0

In the domain of Communication and Autonomy, 93% of women reported none or only a few providers introduced themselves, compared to 82% of providers reporting this. Similarly, 68% of women reported providers never calling them by name or did so only a few times, compared to similar reports by 9% of providers. Providers were also more likely than women to report they involved women in their care, explained the purpose of examinations, procedures, and medicines, and asked permission before procedures. The exception was whether women were allowed to be in a position of their choice during childbirth, where 52% of women reported they were allowed most or all the time, compared to 6% of providers.

In the domain of Supportive Care, 26% of women reported the waiting time was long compared to 18% of providers. Only about 6 and 4% of women reported they were allowed a companion most or all the time during labor and delivery respectively, compared to 12 and 6% of providers, respectively. Women were also less likely than providers to report providers talked to them about their feelings, supported their anxieties, paid attention when they needed help, took the best care of them, did their best to control their pain, and that they completely trusted the providers. About 75% of women felt there were enough staff most or all the time, compared to only 3% of providers; and 37% of women said the facility was crowded most or all the time compared to 42% of providers. Most providers (94%) reported the facility was clean or very clean, compared to very few women (6%). Reports about electricity and safety were similar between groups.

### Qualitative results

The qualitative results focus on providers and are organized by the domains for person-centered maternity care, similar to our quantitative results. The qualitative interviews highlight potential reasons for diverging responses to person-centered care from providers’ perspectives. The results highlight that the discordance in person-centered care is not the result of diverging views, but rather, providers sometimes genuinely not understanding the views of their patients, or justifying their behaviors as necessary.

#### Dignity and respect: rationalizing abuse of women

When discussing whether physical force or verbal abuse occurs in the maternity setting, many providers indicated that this behavior was justified in order to save the life of the baby and mother. Most providers admitted that physical abuse in the labor/maternity ward does occur, but that 1) it happens only rarely, and 2) when it does happen, it is because the provider’s primary responsibility is to ensure that the mom and baby are safe.

“We also become angry … it’s your responsibility to ensure this mother and this baby comes out of that labour ward safe … and this mother is so annoying sometimes … she falls down on the floor, jumping and all those things are risky. So sometimes you also find yourself … overwhelmed even yourself … both psychologically and physically. So you find yourselves just slapping. And sometimes you find it works.” (Maternity provider, female)

“It happens that maybe the mother is here and the baby … and the delivery is taking place. The mother has already pushed the baby and the head is almost out and all over sudden they put the legs together, and you know … she is going to suffocate the baby to death, then try to tell her please don’t, please don’t. Then they refuse to listen, at times you are forced to beat them a little, so that you can get a live baby.” (Maternity provider, female)

Despite some providers explicitly reporting that women perceived their actions as abuse, these providers and staff viewed their behavior as critical to ensuring that they met their main responsibility, which was to keep the woman and baby safe. This represents a difference in the patient/staff-provider perception of physical or verbal abuse.

“I have been in the labor ward for six months, it’s not being insulted, it's being explained to. When a woman comes in to deliver, [and] she feels the child coming out, [yet] she has locked her legs like this, you tell her to open up so that the child can come out but she refuses. What will you do to her? You have to force those legs to be properly placed so that the child can come out, you ask her if you are the one who impregnated her, she wants to kill the child and why would she do that? Won't you be forced to be aggressive? So that she can let the child out … that is how they are told and then they start saying they are talked to rudely yet that is not the case, they are just instructed.” (Support staff, female)

Many providers mentioned that women are not “cooperative” during labor and delivery. Blaming the woman for lack of cooperation provided an opening for providers to explain that verbally or physically abusive behavior was justified in these circumstances. In contrast, a few providers described that mothers that are prepared for labor and childbirth are more cooperative, which may prevent providers from reacting with verbal or physical force.

“Might be this client was not prepared and informed of what to expect during labor. … We do receive some who have never attended antenatal clinic, somebody have just been at home, now she has come. Maybe she has just been carried by a relative brought to the hospital. She doesn’t know what she should expect.” (Maternity provider, female)

“Because sometimes the pain is too much for them, it reaches a point they ... they are not cooperative at all. So that's a major challenge. You find a woman who is very uncooperative ... ok you understand, it’s pain but also with the issues of the pain being too much ... they are also now sometimes you feel it’s like they are overreacting.” (Maternity provider, female)

Providers also indicated that ultimately, the provider is responsible for poor maternal or neonatal outcomes, thus abuse may be justified to ensure favorable outcomes for mother and child or to protect professional status. Some providers also reported that women were grateful for the providers being physically or verbally abusive. These providers recalled how women often thanked the provider for their forceful actions after the baby is born and found to be healthy.

#### Dignity and respect: challenges of assuring privacy and confidentiality

While providers indicated that visual privacy is unequivocally assured, further probing highlighted that privacy is rarely assured 100% of the time. Many providers indicated that privacy is assured for women in their facilities through the use of curtains around beds. In justifying lapses in privacy, providers most frequently cited space, staffing constraints, high patient volume, infrastructure, and the presence of students/other clinicians as barriers to ensuring privacy and confidentiality.

“Yeah in the labour ward there is privacy because we have a screen which we still need to modify to some extent, because even if there is a privacy it’s not a complete … complete privacy … like in our set up there is a mother in first stage, another one in the second stage delivery, so you find that the screen is just screened on one side so this other side … the other mother in first stage can be able to peep and see this person.” (Maternity provider, female)

The majority of providers described the wards as extremely crowded when describing the woman-to-bed ratio. Sharing of beds is common making privacy impossible. Providers also described instances when women were forced to deliver on the floor due to high patient volume and lack of subsequent bed space.

“Just those occasions when they are too many, and they want to deliver at the same time. As you can see, we only have two delivery beds. So if they are four, some will deliver the other side [delivery room.] And the rest this side [waiting room]. It doesn’t matter as long as the child comes out, right?” (Support staff, female)

#### Provider-woman communication: perceptions, challenges, and woman blaming

Many providers indicated that they felt it was their responsibility to give information to women and were adamant about the necessity of doing so. Providers discussed the importance of giving information in order to build rapport, as well as develop and maintain trust with women.

“Let me say it is time, but regardless of even time, me as a clinician, me as a nurse, it’s my obligation. I’m the one who is supposed to inform them.” (Maternity provider, male)

“The one thing … communication, establishment of rapport. For example a patient has come to you, one thing you need to make sure the environment is very conducive … for that patient. … And then from there you can you use a good language for now to approach the patient to be able to identify exactly, what the patient is suffering from and also the confidentiality, privacy … you know there is a way the patients can come in … it becomes very hard for the patient to express him or herself [when there are multiple providers present] … so in that way also you are trying to portray respect because you are creating privacy; you are with this patient alone.” (Maternity provider, female)

Despite acknowledgement of the necessity to provide information to the woman in order to understand her care, providers often described circumstances in which it was acceptable not to do so. Often these circumstances relate specifically to high patient volume and a lack of staff to accommodate the demand, resulting in time constraints that do not allow for providers to give comprehensive counseling to the woman.

“Sometimes you are alone on duty and you are one, and you have like 10 clients, you have five in labor. And you are supposed to monitor the labor, so let me ask you, would you finish all this? You are supposed to give information as in health, hygiene, nutrition, TCA, the baby, the drugs. You cannot do it individually.” (Maternity provider, female)

“Sometimes you can be overwhelmed with [the] job, you [are] very busy, you are all alone, and then a patient wants a lengthy information and then you delay.” (Maternity provider, female)

Blaming women continues to surface as a mechanism to defend lack of provision of information and counseling from the provider’s end. Providers mention that women themselves do not communicate that they need help. Providers also indicate that language can be a significant barrier to providing explanations and information regarding exams or medications. Providers additionally highlight that sometimes women simply don’t follow medical guidance that has been provided to them due to misconceptions or lack of understanding as to the purpose and importance of the directive.

“They’ll ask questions if they have a problem. But … it’s very rare to find a mother just coming to ask [general questions about health]. Because you see a mother is coming to clinic at the age of 24 weeks, that is 6 months. She had never even started even taking even IFAS [iron and folic acid supplementation]. She never even thought it is important, so when she comes that’s when you will inform her. But there [are] very few who will come to ask about the information like that one about the health.” (Maternity provider, male)

“Sometimes [I face barriers] because … I told you we have people from other … parts of the country. Some we will say that they have some issues. … It’s like they are not understanding. Yeah, but we try to use the simple language for them.” (Maternity provider, female)

Despite constraints such as language, time, and staffing, some providers indicated that it was their responsibility to explain what is happening to the woman and/or her family so that women (and especially their families in emergency situations) can make informed decisions.

#### Autonomy: involving/consenting women in their care (woman vs. provider-centered care)

Many providers stated that both involving women in decisions around their care, as well as consenting women is not only important and necessary, but also common. One provider described this by categorizing autonomy as a woman’s right.

“It’s necessary [to involve women and her family in her care] … it will enable the mother and her family to gain confidence, and also generally it’s their right.” (Maternity provider, female)

Many also brought up a formal, written consenting process being done during admission, in which the woman agrees to all procedures that would be done to her throughout her maternity care (including post-partum).

“So that time we have given that episiotomy, you find that that lady, quite a few of them, they urge you that, ‘I don't want be sutured. Let me remain like that.’ So we … can't force them. We usually tell them, we explain to them … we call in their relatives, we explain to them, they talk to her ... it usually takes a procedure. … We have talked to her, she has made her conclusion, we go as per that decision, and then she has to sign. In case of anything, she won't come to blame us.” (Maternity provider, female)

“[The woman is consented] immediately in admission, meaning everything that it will be done to her, she has already agreed on that.” (Maternity provider, female)

While most providers agreed on the importance of involving women in their care, many providers also described examples when providing such autonomy was not employed in order to make prompt decisions during childbirth complications or emergencies.

“But maybe a patient can come already in complications, you don’t have time to [obtain consent] ... so the doctors will sign the consent for them.” (Maternity provider, female)

“I will not ask the mother for consent [if ] … It's like when the mother, she is almost delivering, I can see very well if ... even if I support this mother's perineum in whichever way, she has to get a tear. Okay? She's trying to push it so hard, so that time I will not even ask for her consent to give an episiotomy. I will just give it without even consent … so I better even give that episiotomy, which is simple to repair and to heal, than that mother to get a tear.” (Maternity provider, male)

“[Women will say] ‘I don’t want you to cut me.’ And you tell her, ‘Yes. I will not do it.’ But you’ll do it without her knowledge … Because … we have to ask them in the delivery room if you have realized that this baby is getting tired, the head is just here, the mother she is not fully, she is not well adequate, you cannot say that because she has rejected an episiotomy you’ll not [do it.]. You’ll give [the episiotomy.] Because you know how you give it and you repair it again.” (Maternity provider, male)

Others mentioned not obtaining consent prior to procedures due to workload and fatigue. When asked when consent is not obtained, one provider stated: “I think it’s when maybe the work load is too high. Sometimes you are overwhelmed. Fatigue ... I think those are the times when it’s overlooked.”

#### Supportive care: contradictory perceptions on companion of choice

Provider responses were contradictory on the issue of birth companions. Some providers specify only husbands are allowed, while others indicate that the woman may bring along any companion she would like. Other providers indicated that no one is allowed to accompany a woman in the ward. Among providers who said companions are not allowed, several acknowledged the importance of being able to have a birth companion, although it is still not allowed. Some providers also indicated that there may be exceptions if there is an emergency/referral.

“They can choose whom they want to accompany them because we prepare them and we tell them it’s good you have, we call them birth companions. You have a birth companion, it can be a relative, it can be your spouse, it can be your friend. Yeah, so that one is acceptable depending with what the client chooses.” (Maternity provider, female)

“Unless there is an issue and maybe that patient needs to be referred to another facility, or the doctor wants some information on the relatives, that’s the time the relatives are allowed in maternity.” (Maternity provider, female)

Among providers who responded that women are not allowed to bring a companion, some reflected that there was not enough space in the facility or that the other person could be a distraction, or may facilitate a non-cooperative experience between the woman and provider.

“Like I said we have so many … we admit so many patients such that while we allow them to have partners while giving birth, I think it would really be chaos. So despite the fact that it is important to have a partner, most of the time we discourage it. … I said this to reduce the congestion.” (Maternity provider, female)

Several providers mentioned that others, especially husbands or other men, were not allowed on the ward due to privacy concerns. References to compromised privacy involved the woman herself, other women, or a combination of the two.

“Because you see they are naked. You know when it comes to … that accompaniment, that companionship, at least it needs somewhere there is some privacy, it's the mother and the companion, the two of them only. But in our set-up here you find that it's a big room, one mother is here, the other is there. And the husbands are there, you see, there is no confidentiality.” (Maternity provider, female)

“They can be around, but not in the room because of the issue of privacy in the hospital. I would say they are not allowed but they are supposed to let the patient stay with … anybody they feel comfortable with, but you see maybe the policies, people have different policies. And again because of confidentiality, and privacy. So that is the reason why sometimes they are not allowed, but I feel the choice of the patient or the client is more important.” (Maternity provider, female)

Providers frequently discussed that many women do not desire birth companions. This feedback was corroborated by findings from the survey in which 45% of respondents indicated not wanting someone of their choice with them during their childbirth.

#### Health facility environment: challenges and perceptions

Most providers commented that the maternity wards were clean and that facilities were making concerted efforts to ensure cleanliness. Similar to the privacy issues described above, further probing of providers revealed that they did not perceive the facility to be 100% clean at all times. Providers acknowledged room for improvement, and all of them stressed the critical importance of cleanliness in the labor and postnatal wards for infection control purposes.

“You do this job because you love it, you want a clean place; you want to stay in a clean area and the patients to be in a clean area and the kids too. You know this is a place where there are children.” (Support staff, female)

Providers repeatedly stated barriers to maintaining cleanliness included overcrowding, high patient volume, insufficient staff, and lack of space. A few providers additionally highlighted that the cleaning staff did not always fulfill their duties.

“If the staff who is allocated to do it, she or he is not doing it … water is there in plenty... So if the cleaner is not cooperative, the place can be a mess.” (Maternity provider, female)

Providers additionally blamed women for lack of cleanliness in the washrooms/toilets with some proving to be more empathetic to the women than others.

“Because there are these mothers who would not be able to use the systems the way they are ... they are not conversant with the systems. Others they come from rural areas they are not aware of, so you find that that mess … it can just be improved by talking to the mothers. Telling them how to use them, to take care of their waste ... like others they can just remove the cotton they throw on the floor, others they go you know, pouring blood on the floor.” (Maternity provider, female)

“It depends with how women are … she just does something to make you angry. When she comes and gets someone inside the toilet she just dumps her feces there even in the bathroom, and at that time she waits and says [we always hear them from over here] ‘I’ll just excrete here because there are cleaners.’” (Support staff, female)

## Discussion

Our findings suggest discordance between women and providers’ perspectives in regards to person-centered care experiences. On average, a lower proportion of women report higher levels of person-centered care compared to providers. This includes women reporting low respectful and dignified care, communication and autonomy, and supportive care. Using a mixed-methods approach, we identify two potential reasons that result in this divergence: 1) different understanding or interpretation of behaviors, and 2) different expectations, norms, or values of provider behaviors.

First, provider in-depth interviews shed light on potential scenarios in which providers may understand or interpret behaviors differently than women. A clear example is the different ways that women and providers view and understand the concept of consented compared to non-consented procedures. Quantitative results highlight that almost all providers indicate that they consent women most or all the time, compared to less than half of women who report this. Qualitative interviews suggest that providers may erroneously assume that consent at admission is consent for *all* procedures; therefore, during her care process, while women may not be perceiving that they are being asked permission for different procedures, providers may believe they have done their due diligence in consenting women at admission.

Second, different expectations, norms, or values of providers’ responsibilities may lead to differential reporting of behaviors. For example, across many person-centered care domains, providers suggest that it is part of their job and responsibilities to behave in a certain way, including introducing themselves, explaining medicines, exams, and procedures. Thus, providers may already view these behaviors as part of their job responsibilities and therefore report that person-centered behaviors occur even if women do not view it similarly, or perhaps even if the behavior does not occur. In other instances, providers suggested that it was the woman’s “right” for certain behaviors to occur, including consenting and privacy/confidentiality. If providers view these behaviors as a “woman’s right” they may report these behaviors even if they do not occur in practice. As well, women may not understand underlying provider motivations, which may result in differences in reporting.

It is interesting to note where providers report lower levels of person-centered care in quantitative results. This includes provider reporting higher levels of physical and verbal abuse, lower likelihood of giving women their choice of position during labor, and health facility environment factors such as insufficient staff, crowded labor wards, and lack of water. Past research has suggested that providers justify physical and verbal abuse because their main job responsibility is to save the life of the mother and newborn [13, 19]. Therefore, providers may be more likely to report these behaviors because they are able to justify why it occurs. At the same time, other studies have demonstrated that poor and less empowered women are less likely to report disrespect and abuse [20]. Scholars suggest that this is because poor women expect worse care and therefore do not report negative behaviors when they occur. Our study finds that women may be less likely to report physical and verbal abuse because they perceive it to be normal. It may also be easier for providers to note such abuses as opposed to women because they tend to occur during the second stage of labor, when women are less aware of provider behavior because of the overwhelming pain of childbirth. Moreover, popular press has exposed the high levels of disrespect and abuse in Kenya health facilities; providers, therefore, may be more willing to report that this actually occurs because of this exposure. Facility-level drivers of poor person-centered care included space constraints, which did not allow for privacy or a companion of choice. This is consistent with other studies in Kenya [21].

There are two potential reasons that providers indicate lower levels of person-centered care in regards to health facility environment. First, it does not implicate the providers as many other person-centered care behaviors, and second, providers face significant health facility-related challenges, which may in turn compromise women’s level of care. It is well documented that crowding, overburdened health systems, corruption in health facilities, and lack of human resources all contribute to mistreatment of women [1]. While women may not be aware of these structural challenges, providers are able to report these challenges with more accuracy.

Providers attributed poor person-centered care to both individual and facility/systemic factors. At the individual level, providers blamed women for poor person-centered care. They mentioned women overreacting to pain, not being prepared, or not knowing what to expect when they arrived at the health facility. Other providers discussed women not asking questions because their health literacy levels were low. If true, this could partially explain the differences between provider and woman viewpoints regarding being able to ask questions of providers: providers may be willing to answer questions, but if women are not equipped with health literacy to ask questions, they will not feel able to ask questions. However, other research with women in Kenya suggests that provider attitude is a key reason why some women do not ask questions, with women stating they don’t feel able to ask questions because of fear of how the providers will respond [21]. Research also suggests that higher levels of concordance of social characteristics, such as age, gender, educational level, and socioeconomic status, between patients and providers result in higher satisfaction with care [22]. Therefore, future studies should examine how social concordance in regards to education level and status may lead to greater levels of mutual understanding between patients and providers. In other instances, providers suggested that abuse towards women was in reaction to women abusing providers. It is important for future studies to understand this bi-directional relationship.

This study has a number of limitations. First, the small sample of providers limits the generalizability of our findings and makes this study exploratory in nature. Consequently, we do not provide tests to assess statistical significance across women and providers. Future studies should assess person-centered care at a larger scale among providers. Second, while the person-centered care measures have been validated for use with women, they have not been validated for the provider population. Providers were asked about person-centered care behaviors in general; the questions were not centered on their own behaviors or on specific patient-provider events within a limited period. Thus, they could respond based on any encounter with any provider in any time period. Third, the differences in responses may be due to social desirability bias. Despite our emphasis in the consenting process that anything respondents shared will not be shared with their supervisors or be used to assess their performance, providers may be responding in a socially desirable manner given their employment at these facilities, and the need to project a positive image about the facility, their colleagues, and themselves. Similarly, we assured women that their responses would not be shared with the providers or affect their future care in any way, but women may be also responding in a socially desirable manner because they are answering questions at the facility. Additionally, women’s perceptions of their birth experience within a few days of birth are influenced by their birth outcomes. One study found that approximately 20% of women reported some form of disrespect and abuse when they are at the health facility, but when they were re-interviewed at home, over 28% reported some form of abuse [2].

Another potential limitation is that in some instances, facility staff helped researchers identify and recruit women who may be eligible to participate in the study. It is possible that providers identify women who they perceived as experiencing better care. We would expect that this would result in higher reports of positive person-centered care. Moreover, the data collection for providers and patients differed slightly by a few months; however, the research team recorded major events across the project life span that might impact quality improvement and no major changes occurred during this time. Lastly, health facilities were chosen as part of a larger quality improvement project. Therefore, these health facilities may be more motivated to improve their facilities than other government hospitals. Other hospitals, therefore, might report even higher levels of poor person-centered care and discordance in patient/provider responses. Despite these limitations, this study is unique in that we are able to compare and contrast patient and provider perspectives related to care that women receive.

## Conclusions

This study goes beyond the qualitative explorations of disrespect and abuse with women and providers to quantitatively assessing differences in reports of PCMC, and then explores reasons for the high discordance from providers’ perspectives. We find that most of the responsibility for the discordance lies with providers, the more powerful person in the patient-provider pair, and the one with more agency in their interaction. The findings provide four clear and actionable directions for improving quality of care for women. First, because of the high discordance between woman and provider perspectives, providers should be trained on examining women’s needs and values, in addition to counseling women to be involved in their care. The PCMC scale may help providers understand behaviors that are most important for improving women’s experiences during maternity care. Training on how to obtain permission and consent throughout the care process, as well as alternative ways of gaining patient cooperation that do not involve verbal or physical abuse, are also needed. Second, women should be counseled on their rights and what to expect during labor and childbirth. Additionally, women should be counseled on strategies for engaging with their providers. Lastly, ensuring adequate health systems factors such as the facility environment, human resources, and adequate provider training and support are critical to promoting a person-centered care approach.

## Abbreviation

PCMC: Person-centered maternity care
